# The Role of microRNA in Head and Neck Cancer: Current Knowledge and Perspectives

**DOI:** 10.3390/molecules19055704

**Published:** 2014-05-05

**Authors:** Giulia Courthod, Pierfrancesco Franco, Loredana Palermo, Salvatore Pisconti, Gianmauro Numico

**Affiliations:** 1 Medical Oncology Department, AUSL Valle d’Aosta, Aosta Postcode 11100, Italy; E-Mails: g.courthod@hotmail.it (G.C.); gnumico@ausl.vda.it (G.N.); 2 Radiation Oncology Department, AUSL Valle d’Aosta, Aosta Postcode 11100, Italy; E-Mail: pfranco@ausl.vda.it; 3 Medical Oncology Unit—National Cancer Research Centre Istituto Tumori “Giovanni Paolo II”, Bari Postcode 70124, Italy; 4 Medical Oncology Unit—S.G. Moscati Hospital ASL TA/1, Taranto Postcode 74100, Italy; E-Mail: salvatorepisconti@hotmail.it

**Keywords:** microRNA, hand and neck cancer, biomarkers, chemoresistence, radioresistence

## Abstract

Head and neck cancer is one of the most commonly diagnosed malignancies worldwide. Patients with advanced disease stages frequently develop recurrences or distant metastasis, which results a five-year survival rates of less than 60% despite considerable advances in multimodality therapy. A better understanding of molecular basis of tumorigenesis is required to improve clinical outcomes and to develop new anti-cancer drugs. microRNAs (miRNAs) are a class of small, non-coding, RNA molecules that modulate gene expression post-transcriptionally. They are important regulator in normal biological process; however miRNAs deregulation has been observed in many different tumors and is involved in tumorigenesis. miRNAs may act as tumor suppressors or as oncogenes. Several studies on head and neck cancer demonstrated how aberrant expression of miRNAs is involved in proliferation, metastasis, chemoresistence, and radioresistance. In addition, miRNAs are excellent biomarker targets because they circulate stable in human body fluids and can be obtained with non-invasive methods. Moreover, miRNAs up and down regulation has been correlated with specific cancer phenotype (poor prognosis, aggressiveness and resistance to treatment), playing a role as prognostic biomarkers. This review summarizes current finding on miRNAs in head and neck cancer and their potential role as target for next drug therapy.

## 1. Introduction

Head and neck cancer (HNC) is a heterogeneous oncological setting arising from oral and nasal cavities, pharynx or larynx. It is the sixth most common cancer worldwide. The incidence of HNC in the European Union for 2012 was estimated in 73,014 new cases/year: Germany, France and Denmark showed the highest risk [1]. Squamous cell carcinoma is the most common histologic type (more than 90% of cases). Adenocarcinoma, basal cell and small cell, lymphomas, sarcomas and others are uncommon. Chronic tobacco, alcohol abuse and Human Papilloma-Virus (HPV) infection are the main risk factors for the development of squamous-cell HNC (SCHNC). In patients presenting with early, localized disease, surgery or radiotherapy achieve cure in the large majority of cases. Locally advanced disease is usually treated using integrated treatment modalities including chemotherapy with a worse long-term prognosis. Patients with loco-regional relapse or metastatic disease might benefit from palliative chemotherapy but usually cannot be cured.

Inactivation of tumor suppressor genes (e.g., p53, p16inK4a), activation of proto-oncogenes (e.g., cyclin D, Rb) and enhanced expression of the epidermal growth factor receptor (EGFR) have been observed in SCHNC [2]*.* Cetuximab, an anti-EGFR monoclonal antibody, has demonstrated activity when combined with radiotherapy in locally advanced disease [3] and in combination with chemotherapy in the relapsed/metastatic setting [4], and has been approved for the treatment of SCHNC [5]. On-going clinical trials are evaluating the efficacy of others EGFR monoclonal antibodies (panitumumab, zalutumumab) and EGFR tyrosine kinase inhibitors (gefitinib, lapatinib, afatinib, erlotinib) in these patients [6]. Despite the improvement of surgery, chemotherapy and radiotherapy, the 5-year survival rates are less than 60% [7]. In the last years, increasing interest has been focused on the role of microRNA (miRNA) in cancer. As in others malignancies, miRNA regulates several oncogenes and tumor suppressors, driving the growth, proliferation, metastatic attitude and drug resistance also.

## 2. miRNA

miRNAs are a large family of high conserved non-coding, double-stranded RNA, consisting of about 18–25 nucleotides, that are able to regulate expression of multiple target genes in normal biological process (such as proliferation, differentiation and apoptosis) at post-transcriptional level. To date, 1,872 different miRNAs have been identified in human and the number is still rising [8]*.*

**Figure 1:**
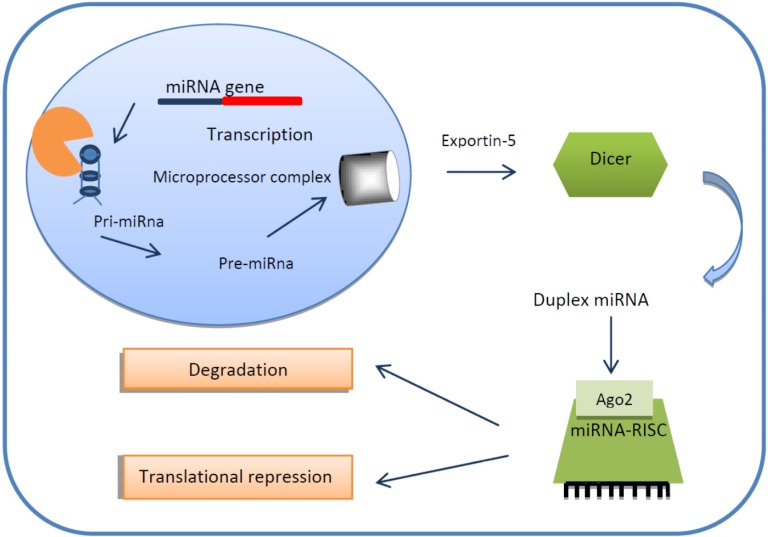
MiRNA biogenesis. MiRNAs genes are transcribed by RNA polymerase into primary miRNAs (pri-miRNA). They are converted into second precursors (pre-miRNA) by the Microprocessor Complex, composed by DiGeorge Syndrome Critical region 8 (DGCR8) and Drosha ribonuclease (RNase III). They are then exported to the cytoplasm by Exportin 5 and are processed into mat ure miRNA/miRNA duplex by DICER enzyme. One of the strands is combined with Argonaute proteins (Ago2) to RNA Induced Silencing Complex (RISC). This complex mediates gene expression by binding target mRNAs and inducing mRNA degradation.

miRNA genes are transcribed by RNA polymerase II (Poll) to form primary miRNA (pri-mRNA) characterized by a specially modified nucleotide at the 5ʹend and a polyadenilated at the 3ʹend (Figure 1). The Microprocessor Complex, formed by a nuclear protein known as DiGeorge Syndrome Critical region 8 (DGCR8) and an enzyme called Drosha ribonuclease (RNase III), convert pri-mRNAs into a second precursor (pre-miRNA) [9,10]. This is then transported into cytoplasm through Ran-GTP-dependent transporter Exportin-5 where it is cleaved by the RNase III enzyme Dicer to create a miRNA-miRNA duplex. In order to form the final complex that performs gene silencing, the double-strained needs to be separated into the functional strand and loaded together with Argonaute proteins into the RNA-induced silencing complex (RISC). This complex mediates gene expression by binding target mRNAs and inducing mRNA degradation [11]*.* Several studies reported aberrantly expression of miRNAs in several cancers, where they play a role as tumor suppressors or as oncogenes. Up-regulation of oncogenic miRNAs results in down-regulation of tumor suppressor genes whereas down-regulation of tumor suppressive miRNAs results in up-regulation of oncogenes [12]*.* Endogenous circulating miRNAs are stable, and are protected from RNases, remaining steady even after being subjected to unfavourable conditions [13].

miRNAs were discovered in 1993, within Ambros and Ruvhun’s lab, on *Caenorhabditis elegans*. An intense research started in the following years aimed at understanding miRNAs functions and possible applications. miRNAs are implicated in several biological and pathologic processes. Great effort is focused on explanation of the potential role of miRNAs in inflammatory disease, atherosclerosis, metabolic disease and fibrosis [14]. They are also important regulator of immune system and angiogenesis. Moreover, miRNA involvement in tumorigenesis was demonstrated by the development of lymphoproliferative tumors in transgenic mouse whose miR-155 gene was up-regulated [15]. As in hematologic neoplasms, solid tumors show aberrant expression of several miRNAs.

## 3. miRNA Deregulation in Head and Neck Cancers

A summary of recent studies regarding miRNA expression profiling in SCHNC is shown in Table 1 [16,17,18,19,20,21,22,23,24,25,26,27,28,29,30,31,32,33,34,35,36,37]. The vast majority of studies included a slender number of cases and informations are not conclusive yet. miR-21 has been shown to act as an oncogene by targeting PTEN in various cancer [38,39]. The miR-21 has also been frequently observed in HNC as reported in Table 1. Recent studies on SCHNC demonstrated that increased expression of miR-21 causes reduction of PTEN expression [27] and its transcriptional regulator Grhl3 [40]. PTEN is an inhibitor of the PI3K pathway. Its inactivation leads to accumulation of phosphatidylinositol 3,4,5-triphosfate (PIP_3_) and consequently increased activity of serine/threonine protein kinase PDK-1 and AKT which promote cell cycle progression, proliferation and inhibit apoptosis. miR-31 activates the hypoxia-inducible factor (HIF) pathway through the suppression of its inhibitors factors (FIH), consequently promoting tumor angiogenesis and growth. As in other cancers, deregulation of miR-31 was observed in SCHNC [28,36]. miR-375 is frequently found as down-regulated in SCHNC and acts as tumor suppressor. Hui *et al.* demonstrated that the down-regulation of miR-375, observed in over 91% of HNSCC samples, would result in deregulated proliferation, chaotic growth and inhibited apoptosis [24]. Moreover, Nohata *et al.* suggested that miR-375 acts as tumor suppressor and regulates AEG1/MTDH (astrocyte elevated gene 1/metadherin), a mediator in different signaling pathways such PI3k/AK, NF-Kb and Wnt/b-catenin, promoting oncogenesis [30].

**Table 1:** miRNA expression profiling in SCHNC recent studies.

Reference	Materials	Methods	miRNA Deregulation
Avissar *et al.* [16]	16 tumors, 5 normal	microRNA array	↑ miR-21, miR-181d, miR-181b, miR-491, miR-455, miR-455, miR-18a, miR-130b, miR-221, miR-193b, miR-181a, miR-18b ↓ miR-375
Barker *et al.* [17]	12 primary tumors, 12 metastatic lymphnodes	TaqMan microRNA assay	↑ miR-103, miR-155, miR-191, miR-181b, miR-181d, miR-205
Cao *et al.* [18]	60 tumors, 54 normal	microRNA array	↑ miR-125, miR-145 ↓ miR-93, miR-205, miR-21, miR-708
Cervigne *et al.* [19]	12 tumors, 7 normal	TaqMan microRNA assay	↑ miR-21, miR-181b, miR-345, miR-146a, miR-184, miR-518b, miR-520g, miR-649
Chang *et al.* [20]	8 tumors, 4 normal	microRNA array	↑ miR-21, miR-155, miR-146, miR-29c, miR-18, miR-146b, let-7i, miR-142-3p ↓ miR-494
Childs *et al.* [21]	8 tumors, 8 normal	MicroRNA array	↑ miR-21, miR-24, miR-151, miR21, miR-199b ↓ let-7f, miR-142-3p, miR-324-5p, miR-368, miR-370, miR-373, miR-422b, miR-424, miR-95, let-7a, miR-16-2, miR-1, miR-133a
Christensen *et al.* [22]	513 tumors, 597 normal	TaqMan microRNA assay	↓ Let-7 family
Fletcher *et al.* [23]	19 tumors, 7 normal	qRT-PCR	↑ miR-205
Hui *et al.* [24]	51 tumors, 4 normal	TaqMan microRNA assay	↑ miR-423, miR-93, miR-106b, miR-16, miR-20a, miR-155, miR-193a, miR-25, miR-92, miR-17-5p, let-7i, miR-19b, miR-223, miR-27a, miR-142-3p, miR-210, miR-106a, miR-15a, miR-21, miR-29b, miR-130b, miR-205, miR-422b ↓ miR-125b, miR-375, miR-10a, let-7a, miR-140, miR-100, miR-143, miR-99a, miR-30c, miR-365, miR-127, let-7c, miR-199b, let-7e, miR-26a
Kozaki *et al.* [25]	18 tumors, 1 normal	qRT-PCR	↑ miR-374, miR-340, miR-224, miR-10a, miR140, miR-181a ↓ miR-27a, miR-34b, miR-34c, miR-203, miR-302c, miR-23a, miR-27b, miR-34a, miR-215, miR-299, miR-330, miR-337, miR-107, miR-133b, miR-138, miR-139, miR-223, miR-204, miR-370, let-7d, mir-95, miR-302a, miR-367*
Lajer *et al.* [26]	49 tumors, 39 normal	microRNA array	↑ miR-31, miR-21, miR-223, miR-503, miR-187, miR-1246, miR-146b-5p, miR-146a, miR-155, miR-424, miR-181a, miR-181b, miR-27a, miR-132, miR-106b, miR-345, miR-21 ↓ miR-375, miR-1224-5p, miR-617, miR-99a, miR-125b, miR-378, miR-27b, miR-125b-2
Li *et al.* [27]	10 tumors, 10 normal	microRNA array	↑ miR-21
Liu *et al.* [28]	10 tumors, 10 normal	TaqMan microRNA assay	↑ mir-31, miR-34c, miR-187, miR-135b, miR-372, miR-34b, miR-21, miR-371, miR-216, miR-301, miR-10a, miR-155, miR-130b, miR-223, miR-373, miR-96, miR-224, miR-147, miR-128b, miR-104, miR-183, miR-182 ↓ miR-100, miR-328, miR-99a, miR-124, miR-149, miR-139, miR-124a, miR-204, miR-211
Kikkawa *et al.* [29]	10 tumors, 10 normal	TaqMan microRNA assay	↑ miR-517c, miR-196a, miR-7, miR-196b, miR-650, miR-18a, miR-452, miR-183, miR-432, miR-301a, miR-21 ↓ miR-1, miR-375, miR-139-5p, miR-504, miR-125b, miR-199b, miR-100, miR-497, miR-30a, let-7c, miR-218, miR-10b, miR-126, miR-378, miR-328, miR-204, miR-143, miR-126, miR-99a, miR-195, miR-489, miR-203, miR-140-5p, miR-29a, miR-26a, miR-214, miR-30a, miR-26b, miR-30e, miR-30b, let-7b
Nohata *et al.* [30]	5 tumors, 5 normal	TaqMan microRNA assay	↓ miR-874, miR-133a, miR-375, miR-204, miR-1, miR-139-5p, miR-145, miR-143, miR-486-3p, miR-146a, miR-410, miR-126, miR-539, miR-134, miR-218, miR-146b-5p, miR-140-3p, miR-30a-3p, miR-191, miR-186, miR-148a, miR-30e-3p, miR-29c*
Ramdas *et al.* [31]	5 Tumors, 5 normal	microRNA array	↑ miR-7083, miR-7, miR-34b, miR-155, miR-182, miR-21, miR-181c, miR-181a, miR-25, miR-93, let-7i, miR-107, miR-103, miR-221 ↓ miR-23b, miR-7029, miR-125a, miR-125b
Rentoft *et al.* [32]	21 tumors, 8 normal	microRNA array	↑ miR-424, miR-21, miR-1301, miR-7, miR-142-5p, miR-105, miR-142-3p, miR-146b-3p, miR-659, miR-361-3p, miR-665, miR-146 b-5p* ↓ miR-617, miR-29b-2, miR-132, miR-548b-5p, miR-22, miR-629, mir-29b-1, miR-99a, let-7c*
Scapoli *et al.* [33]	15 tumors	microRNA array	↑ miR-489, miR-129, miR-23a, miR-214, miR-23b, miR-92, miR-25, miR-210, miR-212, miR-515, miR-146b, miR-21, miR-338 ↓ miR-520h, miR-197, miR-378, miR-135b, miR224, mir-34a
Tran *et al.* [34]	9 Tumors	microRNA array	↑ miR-21, miR-205, miR-16, let-7a ↓ miR-342, miR-346, miR-373
Wiklund *et al.* [35]	15 tumors, 7 normal	qRT-PCR	↑ miR-127, miR-200, miR-205 ↓ miR-375, miR-137
Wong *et al.* [36]	4 tumors, 4 normal	TaqMan microRNA assay	↑ miR-184, miR-34c, miR-137, miR-372, miR-124a, miR-21, miR-124b, miR-31, miR-128a, miR-34b, miR-154, miR-197, miR-132, miR-147, miR-325, miR-181c, miR-198, miR-155, miR-30a-3p, miR-338, miR-17-5p, miR-104, miR-134, miR-213 ↓ miR-133a, miR-99a, miR-194, miR-133b, miR-219, miR-100, miR-125b, miR-26b, miR-138, miR-149, miR-195, miR-107, miR-139
Xiao *et al.* [37]	20 oral leukoplakia, 7 malignant transformed oral leukoplakia	microRNA assay	↑ miR-31, miR-142-5p, miR-33a, miR-1259, miR-146b-5p, miR-886-3p, miR-886-5p, miR-519d, miR-301a ↓ miR-572, miR-611, miR-602, miR-675, miR-585, miR-623, miR-637, miR-1184

## 4. miRNAs as Prognostic Indicators

The prognosis of advanced SCNHC is dismal despite improvements in multimodal treatment. Recent studies showed a potential role of miRNAs as prognostic biomarkers.

Let-7 is a family of tumor suppressing miRNAs whose levels were found deregulated in SCHNC by different authors ([21,22,23,24,25,26,27,28,29,30,31,32,33] and others). Moreover Scapoli *et al.* [33] found a correlation between underexpression of Let-7, miR-155 and miR-146a and progression to metastatic tumors.

In a study on nasopharyngeal carcinoma cells, Wong *et al.* found low levels of Let-7 miRNA and suggested a role in regulating proliferation through the downregulation of c-MYC expression [41]. Low levels of Let-7 have a proliferative effect on nasopharyngeal cells trough the unsuccessful suppressing effect on c-MYC expression.

Moreover, recent studies on non-small cell lung cancer highlighted the role of Let-7 in the KRAS regulation [42]. A variant allele in the KRAS 3ʹ untranslated region, that binds the let-7 complementary site (KRAS-LCS6), is associated with an increased KRAS expression and low levels of Let-7. Christensen *et al.* demonstrated the presence of KRAS-LCS6 variant in SCHNC and its correlation with poor prognosis, especially in oral cancer [22].

TP53 is a tumor suppressor gene encoding for a protein that regulates the cell cycle. TP53 mutation is a common alteration in cancer with a frequency of 53% found in a large cohort of SCHNC analyzed by Poeta *et al.* TP53 mutations were more frequently in patients with tumors arising from hypopharynx (75%) and larynx (56.7%). Moreover, authors demonstrated a strong correlation between TP53 mutations in SCHNC and high risk of recurrence and poor survival [43]. Ganci *et al.* [44] found a strong association between 49 miRNAs and TP53 status with a particular correlation of a subset of 12 miRNAs with shorter recurrence free-survival whereas 4 of them were associated with lower cancer-specific survival. A correlation between the expression of specific miRNAs, such as miR-375 and miR-210, and outcome of SCHNC patients was indeed reported: low levels of miR-375 correlated with poor survival and distant metastases as high levels of miR-210 correlated with locoregional recurrence [45,46].

## 5. miRNAs as Biomarkers

SCHNC is frequently diagnosed in advanced stage when metastases to regional lymph nodes are already present. Even with combination of surgery, chemotherapy and radiotherapy, these patients have a high risk of recurrence. Thus, an important goal would be the identification of biomarkers for early detection. Recent studies identified a potential role of some miRNAs as diagnostic biomarkers. Actually, miRNAs circulate stably in different human body fluids, such as blood, saliva, urine and breath. Hence they can be accessible with non-invasive methods.

Wong *et al.* detected the presence of miR-184 in the plasma of 80% of patients with tongue squamous cancer (all stages) compared to 13% of healthy patients [36]. In the saliva of patients with oral squamous cell cancer, miR-125a and miR-200 were significantly under expressed compared to controls [47]. Moreover, Liu *et al.* showed high levels of salivary miR-31 in oral cancer while levels of miR-31 in plasma were up-regulated [48,49]. Lastly, Clague *et al.* reported the correlation between one variant allele of miR-26a and an increased risk to develop premalignant oral lesions [50].

## 6. miRNAs and Resistance to Chemotherapy and Radiotherapy

The combination of chemotherapy and radiotherapy is the standard treatment for locally advanced SCHNC. However resistance to anticancer drugs oftenly leads to treatment failure, though alterations of different molecular pathways. Recent studies hypothesized a potential role of miRNAs in chemoresistance. Yu *et al.* found a different expression of miRNAs between cisplatin-sensitive tongue squamous cell carcinoma and cisplatin-resistant sublines: in particular increased levels of let-7 family, miR-23a, miR-214, miR-518c, miR-608 and decreased levels of miR-21 and miR-342 [51]. miR-21 and miR-214 have been already reported to alter the chemosensitivity in others tumors as cholangiocarcinoma and ovarian cancer respectively [52,53]. In this study, miR-214 might induce cell survival and cisplatin resistance through targeting the 3’-untranslated region (UTR) of the PTEN gene, which leads to down-regulation of the protein and activation of Akt pathway.

Several authors have also investigated the role of miRNAs in the development of radioresistance. miRNA expression profiles have been compared in 6 tumors cell lines (3 from gliomas and 3 from squamous cell carcinomas) after ionizing radiation. Levels of miR-24-1, miR-144, let-7i, and miR-1285 were significantly increased following irradiation, confirming their susceptibility to ionizing radiation [54]. miR-205 has been investigated in a radio-resistant nasopharyngeal carcinoma cell line (CNE-2P). High level of miR-205 has been detected compared to cell line control [55]. The 3’-UTR of PTEN contains a binding site for miR-205. PTEN is largely known as an inhibitor of cell cycle progression. Authors suggested that PTEN is responsible for radioresistance through miR-205. Low levels of miR-125b, observed in oral squamous cell carcinoma cells, are correlated with proliferation and radioresistance mechanism, mediated by the downregulation of the intracellular adhesion molecule 2 (ICAM2) [56]. Actually, miR-125b has complementary sequences for ICAM2 mRNA.

## 7. The Application of miRNA to Cancer Treatment

One key possibility for the future is the modulation of altered miRNAs concentration through molecules that replace downregulated miRNAs or using antagonists that binds overexpressed miRNAs. The first clinical trial, using a complementary molecule, was conducted in patients with chronic HCV infection [57]. Miravirsen is a short oligonucleotide that binds and inhibits miR-122. miR-122 is highly expressed in the liver and enhances HCV propagation. An ongoing phase II trial has the purpose to assess the antiviral activity and safety of Miravirsen.

To our knowledge there is only one clinical trial available in cancer patients. MRX34 is a molecule mimicking miR-34, which is found as downregulated in many tumors. miR-34 inhibits cancer proliferation and apoptosis by the deregulation of MYC, MET, BCLR and β-catenin. A phase I ongoing study is evaluating the maximum tolerate dose and the pharmacokinetic of MRX34 in patients with primary liver cancer or liver metastasis [58].

## 8. Conclusions

It is widely recognized that miRNAs are pivotal pawns in the cancer development of many tumors, including HNC. Several studies demonstrated considerable discrepancies in miRNAs levels between tumors and corresponding adjacent normal tissues. Moreover, a change in miRNAs expression has been observed in primary disease compared to metastatic sites, suggesting a role in the tumor progression. miRNAs are easily obtained with non-invasive methods because they circulate stable in blood, urine and saliva. Further studies will be needed to identify specific miRNAs that can be used as biomarkers for early detection or as biomarkers with predictive value. However, to date, there are no data mature enough to introduce miRNAs expression analysis in the current clinical management.

From a therapeutic point of view, miRNA-based therapies are currently in preclinical and clinical development with encouraging results. For miRNAs whose expression is reduced, re-introduction of mimics miRNAs could restore the correct gene targets modulation. Conversely, for miRNAs whose expression is increased, the strategy is aimed to the inhibition through use of anti-miRNAs.
